# An optimized protocol for phenotyping human granulocytes by mass cytometry

**DOI:** 10.1016/j.xpro.2022.101280

**Published:** 2022-04-08

**Authors:** Nora Vivanco Gonzalez, John-Paul Oliveria, Dmitry Tebaykin, Geoffrey T. Ivison, Kaori Mukai, Mindy M. Tsai, Luciene Borges, Kari C. Nadeau, Stephen J. Galli, Albert G. Tsai, Sean C. Bendall

**Affiliations:** 1 Department of Pathology, School of Medicine, Stanford University, Stanford, CA, United States; 2 Department of Medicine, Division of Respirology, McMaster University, Hamilton, ON, L8S4K1, Canada; 3 Sean N. Parker Center for Allergy Research, School of Medicine, Stanford University, Stanford, CA, United States; 4 Department of Microbiology and Immunology, School of Medicine, Stanford University, Stanford, CA, United States; 5 Department of Medicine, Division of Pulmonary and Critical Care Medicine, School of Medicine, Stanford University, Stanford, CA, United States

**Keywords:** Cell isolation, Single Cell, Flow Cytometry/Mass Cytometry, Health Sciences, Immunology, Antibody

## Abstract

Granulocytes encompass diverse roles, from fighting off pathogens to regulating inflammatory processes in allergies. These roles are represented by distinct cellular phenotypes that we captured with mass cytometry (CyTOF). Our protocol enables simultaneous evaluation of human basophils, eosinophils, and neutrophils under homeostasis and upon immune activation by anti-Immunoglobulin E (anti-IgE) or interleukin-3 (IL-3). Granulocyte integrity and detection of protein markers were optimized so that rare granulocyte populations could be deeply characterized by single cell mass cytometry.

For complete details on the use and execution of this protocol, please refer to Vivanco Gonzalez et al. (2020).

## Highlights

• Mass cytometry antibody panel to deeply characterize granulocyte protein expression
• Peripheral blood processing for optimal granulocyte yield for single cell analysis
• In vitro immune activation to determine granulocytic functional states
• Protocol optimized for detection of rare granulocyte subsets

## Graphical abstract

**Figure:**
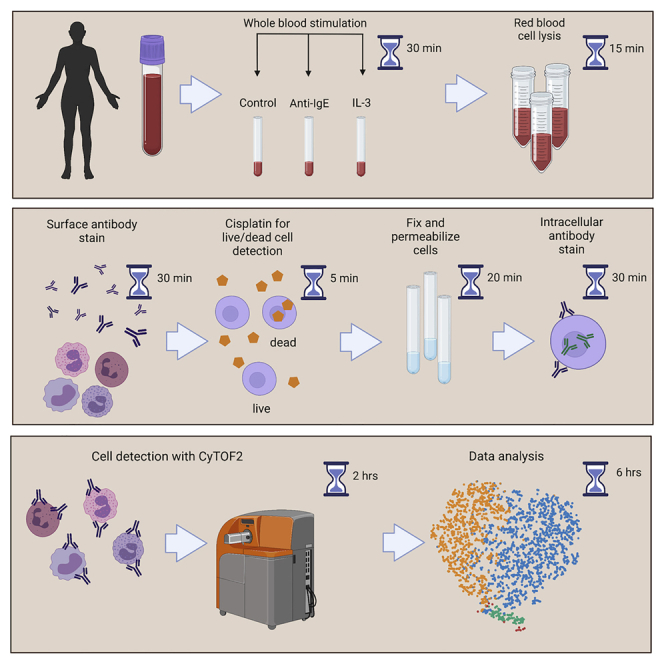

## Before you begin

### Institutional permissions

Permission for human sample acquisition must be granted by the Institutional Review Board (IRB) of your research institution. Peripheral blood samples from anonymous healthy donors (with unknown allergy status) were obtained from the Stanford Blood Center (Palo Alto, CA, USA). Healthy bone marrow and peripheral blood samples paired from the same donor were ordered from AllCells (Alameda, CA, USA). Samples from patients diagnosed with chronic myeloid leukemia (CML) were obtained less than 3 days after collection as post-diagnosis excess material as approved by the IRB of the Stanford School of Medicine.

### Prepare metal-conjugated antibody panel

**Timing: 1 week**

Fluidigm Sciences offers a variety of commercially available antibodies that assess the human immune system, but a substantial portion of antibodies we used were conjugated in house. The conjugation process takes 4 h, and around 4–6 antibodies are conjugated in parallel. The antibodies are then titrated on human peripheral blood that has undergone red blood cell lysis. Depending on the number of antibodies conjugated and accounting for the time used to conduct the antibody titration, the process could take up to a week. For an in-depth protocol on antibody conjugation and titration please refer to Hartmann et al. (2019).

**Figure 1:**
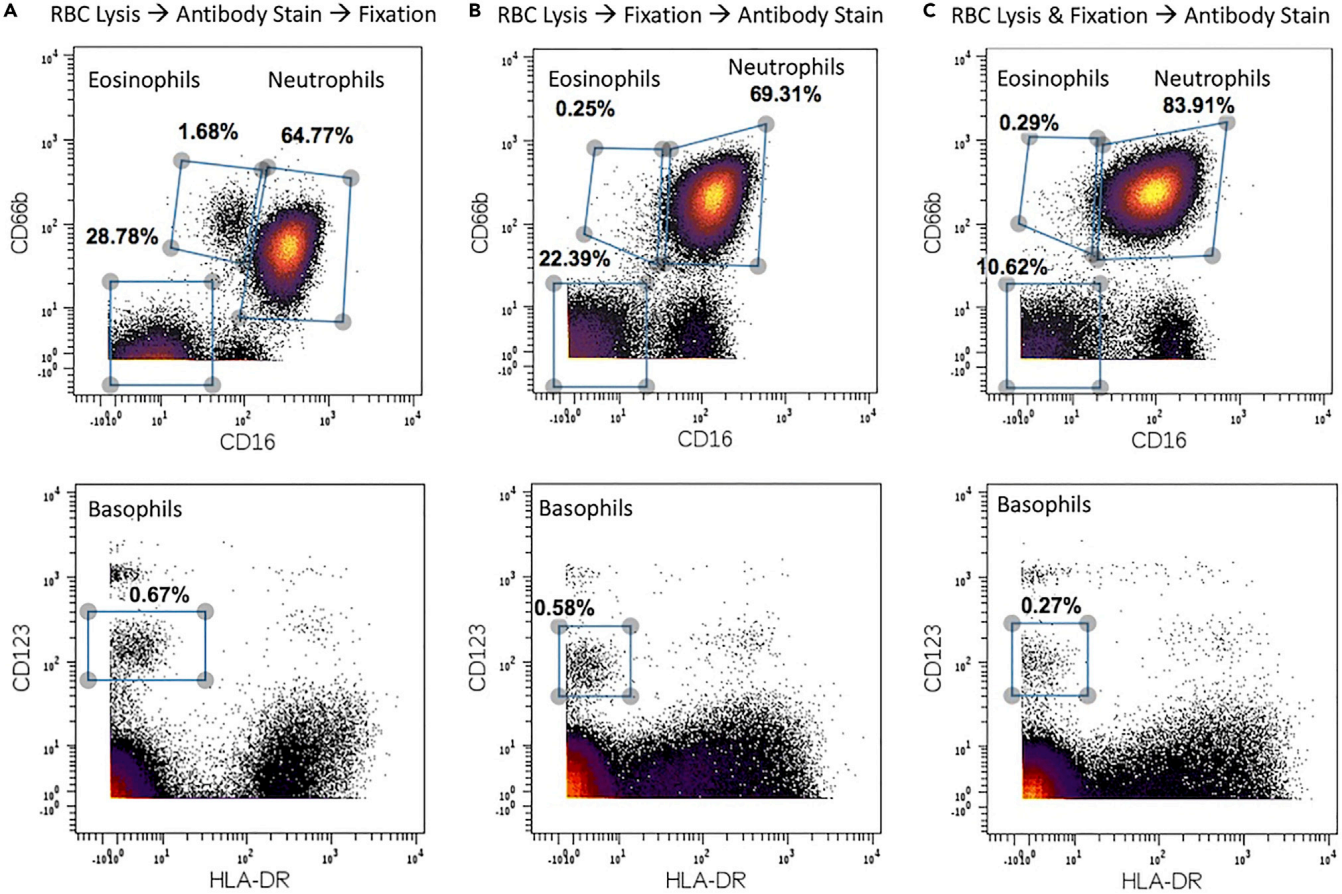
Optimization of eosinophil and neutrophil detection (A) Cells were treated with RBC lysis buffer, followed by staining with surface antibodies (including CD16 and CD66b) prior to fixation with PFA as outlined in protocol. (B) Cells were treated with RBC lysis buffer, fixed with PFA, and then stained with surface antibodies. (C) Cells were treated with a commercially available RBC lysis/fix solution prior to staining with surface antibodies.

1. 200 μg of primary antibodies were conjugated to metal isotopes with the Maxpar X8 Antibody Labeling Kit from Fluidigm Sciences according to Hartmann et al. (2019).
2. After metal conjugation, antibodies were diluted with PBS-based Antibody Stabilization Solution (Boca Scientific) to 0.2 mg/mL and stored long-term at 4°C.
3. An example cocktail used to generate Figure 1 of Vivanco Gonzalez et al. (2020) is available under materials and equipment. We used a mix of commercially available metal-conjugated antibodies and in house conjugated antibodies.
4. The day of the experiment, prepare surface and intracellular antibody cocktails. Keep refrigerated until use.
5. Filter surface and intracellular cocktails with Ultrafree-MC VV 0.1 μm microfiltration centrifugal filter from Millipore.

**CRITICAL:** Antibodies must be purchased in a BSA-free solution to carry out suggested conjugation protocol.

### Additional reagent preparation

**Timing: 1 h**

6. Prepare dPBS + 2 mM EDTA by combining 498 mL cold dPBS + 2 mL 0.5 M EDTA. This can be prepared ahead of experiment. Keep refrigerated until use. Can use up to 1 month before discarding. Sterilize by using an Argos 0.22 μm filter.
7. Filter double-distilled water with an Argos 0.22 μm filter and keep at 4°C until use.
8. The day of the experiment, dilute BioLegend 10× RBC lysis buffer to 1× with double-distilled water. Keep at 4°C until use.
9. Prepare 0.5 mM cisplatin (hazardous) solution: 1 μL of 100 mM cisplatin in DMSO (stored at −20°C) added to 199 μL of low barium PBS. Keep at 4°C until use.
  a. Cisplatin is a hazardous chemical that crosslinks DNA and interferes with cell division. Handle with chemical resistant gloves, safety goggles, face mask, and lab coat, thus minimizing any exposure to skin. Individuals who are pregnant, breast feeding, or attempting pregnancy should not be exposed to this chemical.
10. PFA 16% that has been opened at most 1 month prior to experiment and kept sealed at 20°C–23°C. In total, 1 mL of 16% PFA will be ultimately needed for 1 mL of peripheral blood processed.
11. The day of intracellular staining, prepare DNA intercalator solution.
12. Warm up RPMI 1640 media to 37°C.
13. Prepare fresh, anti-IgE antibody and IL-3 reagents to stimulate granulocytes and keep at 37°C until use:
  a. 1 mL of 2 μg/mL anti-IgE in RPMI: 2 μL of anti-IgE (1 mg/mL) added in 1 mL of warm RPMI. This is a 2× solution.
  b. 1 mL of 4 ng/mL IL-3 in RPMI: 1 μL of recombinant IL-3 (4 ng/μL) added in 1 mL of warm RPMI. This is a 2× solution.

**CRITICAL:** Use sterilized pipette tips with filters for all liquid handling.

## Key resources table

**Table:** 

REAGENT or RESOURCE	SOURCE	IDENTIFIER
**Antibodies**		

CD235ab (Clone HIR2)	BioLegend	Cat#306602
CD45 (Clone HI30)	BioLegend	Cat#304002
CD61 (Clone VI-PL2)	BioLegend	Cat# 336402
CD7 (Clone CD7-6B7)	BioLegend	Cat# 343102
CD294 (Clone BM16)	BioLegend	Cat#350102
CD191 (Clone TG4/CCR1) discontinued	MBL International	Cat#D063-3
Siglec-8 (Clone 7C9)	BioLegend	Cat#347102
CD164 (Clone 67D2)	BioLegend	Cat#324802
CD13 (Clone WM15)	BioLegend	Cat#301702
CD123 (Clone 6H6)	BioLegend	Cat#306002
FcεRI (Clone CRA-1)	BioLegend	Cat#334602
CD11b (Clone ICRF44)	BD	Cat#555386
CD23 (Clone EBVCS-5)	Sigma Aldrich	Cat#SAB4700732-100UG
CD116 (Clone 4H1)	BioLegend	Cat#305902
CD49d (Clone 9F10)	BioLegend	Cat#304302
MRP-14 (Clone MRP 1H9)	Santa Cruz	Cat#53187
CD52 (Clone HI186)	BioLegend	Cat#316002
CD53 (Clone HI29)	BD	Cat#555506
CD305 (Clone NKTA255)	Santa Cruz	Cat#59281
IgE (Clone MHE-18)	BioLegend	Cat#325502
CD203c (Clone NP4D6)	BioLegend	Cat#324602
CD244 (Clone C1.7)	BioLegend	Cat#329502
CD88 (Clone S5/1)	BioLegend	Cat#344302
CD71 (Clone CY164)	BioLegend	Cat#334102
CD105 (Clone 43A3)	BioLegend	Cat#323202
proMBP1 (Clone J175-7D4)	BioLegend	Cat#346802
CD56 (Clone NCAM16.2)	BD	Cat#559043
MPO (Clone 1B10)	BD	Cat#556035
rRNA (Clone Y10b)	Novus Biologicals	Cat#NB100-662
CD38 (Clone HIT2)	BioLegend	Cat#303502
CD117 (Clone 104D2)	BioLegend	Cat#313202
Ki67 (Clone B56)	BD	Cat# 550609
CD64 (Clone 10.1)	BioLegend	Cat#305002
CD3 (Clone S4.1) QD655, contains cadmium (112/114)	Invitrogen	Cat#Q10012
HLA-DR (Clone L243) -174Yb	Fluidigm Sciences	Cat#3174001B
CD193 (Clone 5E8) -175Lu	Fluidigm Sciences	Cat#3175025B
CD44 (Clone IM7) - 171Yb	Fluidigm Sciences	Cat#3171003B
CD15 (Clone W6D3) - 164Dy	Fluidigm Sciences	Cat#3164001B
CD14 (Clone M5E2) - 160Gd	Fluidigm Sciences	Cat#3160001B
CD33 (Clone WM53) - 158Gd	Fluidigm Sciences	Cat#3158001B
CD183 (Clone G025H7) - 156Gd	Fluidigm Sciences	Cat#3156004B
CD66b (Clone 80H3) - 152Sm	Fluidigm Sciences	Cat#3152011B
CD20 (Clone 2H7) - 147Sm	Fluidigm Sciences	Cat#3147007B
CD16 (Clone 3G8) - 148Nd	Fluidigm Sciences	Cat#3148004B

**Biological samples**		

Peripheral blood from healthy donors in EDTA or heparin	Stanford Blood Center	stanfordbloodcenter.org
Paired bone marrow and peripheral blood from healthy donors	AllCells	https://www.allcells.com/
CML samples	Obtained under informed consent with IRB approval	N/A

**Chemicals, peptides, and recombinant proteins**		

RPMI 1640 media	Thermo Fisher Scientific	Cat#11879020
Human TruStain FcX (Fc Receptor Blocking Solution)	BioLegend	Cat#422302
Cell-ID Intercalator-Ir	Fluidigm Sciences	Cat#201192A
Cisplatin (hazardous)∗	Sigma-Aldrich	Cat# P4394-25MG
Calibration Beads, EQ, Four Element	Fluidigm Sciences	Cat#201078
Paraformaldehyde (PFA) 16%, at 20°C–23°C	Electron Microscopy Sciences	Cat#50-980-489
Polyclonal rabbit anti-IgE	Bethyl Laboratories	Cat#A80-109A
IL-3, −20°C	PeproTech	Cat#200-03
10× RBC lysis buffer	BioLegend	Cat#420301
EDTA	Invitrogen Life Technologies	Cat#15575020
PBS-based Antibody Stabilization Solution	Boca Scientific	Cat#131 050

**Critical commercial assays**		

Maxpar X8 Antibody Labeling Kit, −20°C	Fluidigm Sciences	Cat#201300
Ultrafree-MC VV Centrifugal Filter	Millipore	Cat#UFC30VV00
Argos Technologies Disposable Bottle Top Aspirator System, PES Membrane, 0.22 um, 500 mL	Cole Parmer	Cat#EW-07630-06
EQ Four Element Calibration Beads	Fluidigm Sciences	Cat#201078
Falcon Round-Bottom Polystyrene Test Tubes with Cell Strainer (35 μm) Snap Cap	Fisher Scientific	Cat#08-771-23

**Software and algorithms**		

Cytobank	Cytobank Inc.	http://cytobank.org
R	R Core Team (2017)	http://www.r-project.org
Scanpy	Wolf et al. (2018)	https://doi.org/10.1186/s13059-017-1382-0
Python	N/A	http://www.python.org
MATLAB-based bead normalization software	Finck et al. (2013)	https://doi.org/10.1002/cyto.a.22271

Note: Antibody dilutions can be found in Tables 1 and 2. Unless otherwise stated, reagent kept at 4°C.

**∗** Cisplatin is a hazardous chemical that crosslinks DNA and interferes with cell division. Handle with chemical resistant gloves, safety goggles, face mask, and lab coat, thus minimizing any exposure to skin. Individuals who are pregnant, breast feeding, or attempting pregnancy should not be exposed to this chemical.

***Alternatives:*** Cell-ID Cisplatin from Fluidigm Sciences (Cat#201064) can be used instead of Cisplatin. The anti-CD191 (Clone TG4/CCR1) antibody by MBL International (Cat#D063-3) has been discontinued, but our lab has successfully used anti-CD191 (Clone 5F10B29) by BioLegend (Cat#362902) in other antibody panels. Any antibodies available from Fluidigm Sciences can be used to replace antibodies that we conjugated in house. Antibody efficacy is further discussed in the troubleshooting section. The premessa R package (ParkerICI/premessa) can be used instead of the MATLAB-baes bead normalization software.

**Table 1:** Mass cytometry surface antibody panel

Antigen	Element symbol	Isotope Mass	Stock conc. (mg/mL)	Desired final conc. (μg/mL)	Vol. per 100 μL reaction (μL)	Cocktail mix Vol.+ 10%
CD3 (QD655)	Cd	112/114	0.2	3	1.5	4.95
CD235ab	In	113	0.2	1	0.5	1.65
CD45	In	115	0.1	1	1	3.3
CD61	La	139	0.2	0.5	0.25	0.825
CD7	Pr	141	0.2	2	1	3.3
C3aR	Nd	142	0.2	1	0.5	1.65
CD294	Nd	143	0.2	2	1	3.3
CD191	Nd	144	0.2	4	2	6.6
Siglec-8	Nd	145	0.2	4	2	6.6
CD164	Nd	146	0.2	4	2	6.6
CD20	Sm	147	0.2	2	1	3.3
CD16	Nd	148	0.2	2	1	3.3
CD69	Sm	149	0.075	0.75	1	3.3
CD13	Nd	150	0.2	1	0.5	1.65
CD123	Eu	151	0.2	2	1	3.3
CD66b	Sm	152	0.2	2	1	3.3
FcER1	Eu	153	0.2	1	0.5	1.65
Siglec-10	Sm	154	0.2	2	1	3.3
CD11b	Gd	155	0.2	2	1	3.3
CD183	Gd	156	0.2	2	1	3.3
CD23	Gd	157	0.2	8	4	13.2
CD33	Gd	158	0.1	1	1	3.3
CD116	Tb	159	0.2	2	1	3.3
CD14	Gd	160	0.2	2	1	3.3
CD49d	Dy	162	0.2	1	0.5	1.65
CD15	Dy	164	0.2	2	1	3.3
CD107	Ho	165	0.2	4	2	6.6
CD52	Er	166	0.2	1	0.5	1.65
CD53	Er	167	0.2	3	1.5	4.95
CD305	Er	168	0.2	1	0.5	1.65
IgE	Tm	169	0.2	1	0.5	1.65
CD63	Er	170	0.2	2	1	3.3
CD44	Yb	171	0.2	2	1	3.3
CD203c	Yb	172	0.2	0.5	0.25	0.825
CD244	Yb	173	0.2	1	0.5	1.65
HLA-DR	Yb	174	0.2	2	1	3.3
CD193	Lu	175	0.2	4	2	6.6
CD88	Yb	176	0.2	4	2	6.6
				Total (μL)	42	138.6
				CSM	10.5	34.65
				Total:	52.5	173.25
					Per Test	Full cocktail volume (μL)

Surface antibody cocktail for 3 tests (control, anti-IgE, IL-3), each having around 10 million cells. CSM stands for Cell Staining Media. The final staining volume per test is 100 μL, 52.5 μL of antibody cocktail plus 47.5 μL of cell suspension sample.

Note: All antibodies were kept at 4°C.

**Table 2:** Mass cytometry intracellular antibody panel

Antigen	Symbol	Isotope Mass	Stock conc. (mg/mL)	Desired final conc. (μg/mL)	Vol. per 100 μL reaction (μL)	Cocktail mix Vol.+ 10%
Galectin-9	Dy	161	0.2	4	2	6.6
MRP-14	Dy	163	0.2	2	1	3.3
				Total (μL)	3.0	9.9
				CSM	32.0	105.6
				Total:	35.0	115.5
					Per Test	Full cocktail volume (μL)

Intracellular antibody cocktail for 3 tests, each can stain around 10 million cells. CSM stands for Cell Staining Media. Total staining volume is 100 μL, 35 μL antibody cocktail plus 65 μL of cell suspension sample.

Note: All antibodies were kept at 4°C.

## Materials and equipment

**Table:** 10× low barium PBS preparation

Reagent	Final concentration or percentage	Amount
NaCl	8% wt/vol	320 g
KCl	0.2% wt/vol	8 g
Na_2_HPO_4_·7H_2_O	1.15% wt/vol	46 g
KH_2_PO_4_	0.2% wt/vol	8 g
ddH_2_O	n/a	3 L
**Total**	**n/a**	**4 L**

Note: Store low barium PBS at 4°C for at most 3 months.

**CRITICAL:** Bring solution to pH 7.4 using concentrated aqueous NaOH. Bring volume to 4 L with double distilled water. Sterilize by using an Argos 0.22 μm bottle top filter. To create a 1× low barium PBS, mix one part of 10× stock with nine parts double distilled water.

***Alternatives:*** To maintain the CyTOF’s sensitivity, solutions used in the cell immunostaining protocol should have less than 1 ppb of barium. Commercially available dPBS has high barium contamination and should be avoided in the latter steps of cell staining and washing.

**Table:** Cell staining medium

Reagent	Final concentration or percentage	Amount
BSA	0.5% wt/vol	2.5 g
Sodium azide	0.02% wt/vol	100 mg
1× low barium PBS	n/a	497 mL
**Total**	**n/a**	**500 mL**

Keep at 4°C for up to 1 month before discarding.

**CRITICAL:** Sodium azide is toxic if consumed, inhaled, or in contact with skin. It should always be handled in a fume hood with chemical resistant gloves, safety goggles, face mask, and lab coat, thus minimizing any exposure. Sterilize cell staining medium by using an Argos 0.22 μm bottle top filter.

**Table:** DNA intercalator solution

Reagent	Final concentration or percentage	Amount
Fluidigm Cell ID intercalator Ir	0.1 μM	1.6 μL
PFA	1.6%	800 μL
1× low barium PBS	n/a	7.2 mL
**Total**	**n/a**	**8 mL**

Prepare fresh minutes before use.

**CRITICAL:** PFA is toxic if consumed, inhaled, or in contact with skin. It should always be handled in a fume hood with chemical resistant gloves, safety goggles, face mask, and lab coat, thus minimizing any exposure.

## Step-by-step method details

### Granulocyte stimulation

**Timing: 2 h**

Whole blood samples were kept refrigerated (2°C–6°C) for up to 24 h by blood collection facility prior to processing as described by Mukai et al. (2017). We used EDTA coated tubes containing a total of 6 mL of blood or heparin coated tubes that contained 10 mL of blood. Mukai et al. describes in detail the effect of time post collection, refrigeration, and anti-coagulant type on CD63 expression post basophil activation test.

Whole blood is stimulated with anti-IgE or IL-3 and processed for downstream cellular analysis.

1. Warm whole blood in 37°C water bath for 30 s.
2. Pipette 1 mL of whole blood into 5 mL snap cap tubes with the following stimulants:
  a. Sample Tube 1: 1 mL of RPMI (control, without stimulation).
  b. Sample Tube 2: 1 mL of 2 μg/mL anti-IgE in RPMI.
  c. Sample Tube 3: 1 mL of 4 ng/mL IL-3 in RPMI.
3. Let sample tubes incubate at 37°C for 30 min in 5% CO_2_ incubator. Make sure tubes are tightly capped.
4. Transfer contents of each tube to corresponding 50 mL falcon tubes.
5. To each tube add 20 mL of cold 1× RBC lysis buffer. Incubate for 15 min in the dark on ice.
6. Stop RBC lysis by adding 20 mL cold dPBS + 2 mM EDTA.
7. Centrifuge cells at 350 × *g*, 5 min, at 4°C. Aspirate most of supernatant, leaving behind approximately 100 μL, in which the pellet of cells can be dissolved by tapping the tube.
8. Add 20 mL dPBS + EDTA to each tube, making sure cells are fully resuspended in fluid by gently tapping tube.
9. Centrifuge at 350 × *g*, 5 min, at 4°C. Aspirate most of supernatant, leaving behind approximately 100 μL, in which the pellet of cells can be dissolved by tapping the tube.
10. Add 3 mL of CSM to each tube and transfer their contents to corresponding FACS tubes.
11. Count cells in hemacytometer post RBC lysis. In each tube, there should be around 10 million cells.
12. Spin tubes for 5 min at 350 × *g*, at 4°C.
13. Carefully aspirate supernatant, leaving residual supernatant to avoid disrupting cell pellet. Tap tube to dissolve cells in solution. Given the concentrations of our antibodies in the panel, the cell volume should be 45 μL per tube prior to adding the surface antibody cocktail. The total staining volume, including surface cocktail will be 100 μL.

**CRITICAL:** Since basophils are a rare cell population, accounting for 1% of leukocytes in peripheral blood, avoid losing cells in washing steps. Decanting the supernatant or trying to aspirate supernatant entirely will result in increased cell loss. Spin live cells at 350 × *g* to maintain cell integrity.

### Blocking and surface immunostaining

**Timing: 1 h**

Cells are prepared and stained with surface antibody markers. The staining cocktail is meant for up to 10 million cells.

14. Add 2.5 μL of Human TruStain FcX (BioLegend) solution. Tap tube to dissolve cells in solution.
15. Incubate for 10 min at 20°C–23°C.
16. Add the volume necessary of the surface antibody cocktail into each tube. For our experiments this was 52.5 μL, bringing the total staining volume to 100 μL.
17. Tap cell volume to resuspend cells and prevent cell clumps.
18. Incubate tubes at 20°C–23°C for 30 min.

### Cisplatin staining and cell fixation

**Timing: 1 h**

Samples are stained with cisplatin (hazardous) to detect dead cells, which must be excluded from analysis. All analyzed cells are live, intact cells. Cisplatin is a hazardous chemical that crosslinks DNA and interferes with cell division. Handle with chemical resistant gloves, safety goggles, face mask, and lab coat, thus minimizing any exposure to skin. Individuals who are pregnant, breast feeding, or attempting pregnancy should not be exposed to this chemical.

19. To each sample tube, add 899 μL of low barium PBS and 1 μL of 0.5 mM cisplatin, for a final concentration of 0.5 μM cisplatin in a total volume of 1 mL.
20. Briefly vortex and incubate for 5 min at 20°C–23°C.
21. Add 4 mL of CSM, and pellet the cells by centrifugation at 250 × *g* for 5 min.
22. Aspirate the supernatant leaving behind approximately 100 μL and resuspend cell pellet by tapping tube.
23. Repeat steps 20–21.
24. To each tube, add 900 μL of CSM buffer and 100 μL of PFA 16% for a final concentration of 1.5% PFA.
25. Incubate for 10 min at 20°C–23°C.
26. Once cells are fixed, pellet the cells by centrifugation at 500 × *g* for 5 min at 4°C.
27. Aspirate the supernatant leaving behind approximately 100 μL and resuspend the cells in the residual volume by tapping the tube.
28. Add 4 mL of CSM. Pellet the cells by centrifugation at 500 × *g* for 5 min at 4°C. Aspirate the supernatant leaving behind approximately 100 μL.

**Pause point:** Fixed cells can remain in CSM suspension at 4°C for 24 h.

**CRITICAL:** Fixing cells after cell surface staining allows for clear distinction of eosinophils from neutrophils when gating with CD16 and CD66b. Otherwise CD16 levels of eosinophils appear indistinguishable from those of neutrophils (Figure 1).

### Cell permeabilization and intracellular staining

**Timing: 1.5 h**

Cells must be permeabilized to permit Intracellular staining with antibody markers.

29. Tapping all sample tubes to resuspend cell pellet, then add 1 mL of 4°C 100% MeOH. Incubate on ice for exactly 10 min.
30. Immediately add 3 mL of CSM to stop further permeabilization.
31. Pellet the cells by centrifugation at 500 × *g* for 5 min at 4°C.
32. Aspirate supernatant, leaving approximately 100 μL of residual volume, as to not disturb cell pellet.
33. Resuspend cell pellet by tapping and repeat steps 29–31.
34. Aspirate supernatant and bring to appropriate volume for intracellular staining. Given our antibody cocktail, we brought the supernatant to a total volume of 65 μL.
35. Add the volume necessary of the intracellular antibody cocktail into each tube. For our experiments this was 35 μL of antibody cocktail, bringing the total staining volume to 100 μL.
36. Incubate for 30 min at 20°C–23°C.
37. Add 3 mL of CSM buffer to each tube.
38. Centrifuge at 500 × *g* for 5 min at 4°C. Aspirate the supernatant leaving approximately 100 μL of residual volume.
39. Resuspend pellet by tapping tube and add 900 μL of DNA intercalator solution to each tube, total volume 1 mL, and gently mix by pipetting up and down.
40. Leave at 4°C for at least 16 h. Alternatively, to run the sample on the CyTOF the same day, leave cells with DNA intercalator solution for 20 min at 20°C–23°C. Place at 4°C until sample is processed.

**Pause point:** Fixed cells in DNA intercalator can remain at 4°C for up to a week prior to running samples through the CyTOF.

### Preparation for acquisition

**Timing: 30 min**

#### Cell preparation for acquisition by CyTOF2

41. Add 4 mL of CSM at 4°C to each sample tube. Resuspend pellet by tapping tube.
42. Centrifuge at 500 × *g* for 5 min at 4°C. Aspirate the supernatant leaving approximately 100 μL of residual volume.
43. Resuspend pellet by tapping tube and add 4 mL of filtered double-distilled water.
44. Centrifuge at 500 × *g* for 5 min at 4°C. Aspirate the supernatant leaving approximately 100 μL of residual volume.
45. Repeat steps 42–43.
46. Dilute EQ Four Element Calibration Beads 10-fold in filtered double-distilled water and keep at 4°C. For example, add 2.5 mL of EQ beads to 22.5 mL double-distilled water to have enough solution for three to four tests. EQ beads are necessary for data normalization, as the instrument signal detection changes slightly over time.
47. Count cells with hemacytometer.
48. Immediately before analysis, resuspend pellet by tapping tube and add diluted EQ beads to reach a cell density of 1 million cells per mL.
49. Filter sample by using a Falcon round-bottom tube with cell strainer (35 μm) to avoid clogging the CyTOF or nebulizer.
50. Cells were acquired at an event rate of less than 500 per second to avoid doublets. If the event rate was higher than 500 per second, the cell solution was further diluted with EQ bead/water mixture.

**CRITICAL:** Keep samples on ice until their acquisition.

### Data analysis

**Timing: 6 h**

After event acquisition, live single cells will be isolated in silico, bead-normalized for changes in instrument signal, and quantile normalized to correct technical variation between CyTOF runs.

51. FCS files for each sample were processed through a MATLAB-based bead normalization software (Finck et al., 2013) before being uploaded to Cytobank for gating.
52. Please refer to Figure 1A of Vivanco Gonzalez et al. (2020) for a detailed gating scheme to isolate live immune cells and specific granulocyte subsets.
53. Individual immune cell types (e.g., basophils) were processed with R (http://www.r-project.org) to quantile normalize protein expression by donor. This approach corrects for technical variation between CyTOF runs.
54. An inverse hyperbolic sine (arcsinh) transformation with a cofactor of 5 was applied to the data before employing Scanpy’s (Wolf et al., 2018) Python-based (http://www.python.org) implementation. Scanpy was used to carry out dimensionality reduction via tSNE and clustering with the Leiden algorithm.

## Expected outcomes

Our approach using whole blood stimulation, live cell staining, and in silico cell enrichment will optimize for detection of rare granulocyte phenotypic states. We suggest starting with at least 1 mL of whole blood from a healthy individual, which yields approximately 10 million cells post red blood cell lysis. Starting with this volume of blood ensures that at least 500,000 events are collected by mass cytometry (CyTOF), leading to robust representation of each granulocyte subpopulation. While phenotypic analysis of prominent granulocyte subtypes can still be conducted in samples with lower cell yields, we suggest collecting at minimum 50,000 events per blood sample. This number of events will permit in depth phenotypic analysis of high abundance cell populations but will likely result in limited representation of basophils and eosinophils. Our protocol includes several steps that ensure careful handling of samples with live granulocytes, since granulocytes are prone to degranulation and in turn cell death. As challenging as it is to process samples with live granulocytes, we do not recommend fixing samples prior to staining cells with surface antibodies. Cell fixation led to an increase in detection of surface CD16 across granulocytes (Figures 1B and 1C), posing a particular challenge in separating eosinophils from neutrophils while using a traditional gating scheme. Before proceeding with phenotypic analysis, bead normalization must be used to eliminate instrument dependent changes in metal isotope detection. Expect to see normal human phenotypic variability between samples. However, before proceeding to identify protein changes across donor samples, quantile normalization of immune cells should be carried out to prevent batch effects in protein marker detection. These batch effects are due to slight variabilities in antibody cocktail preparation, potentially attributed to antibody shelf lives, human error in pipetting antibodies, or the use of an antibody from a different stock. After quantile normalization of protein detection across immune cells, samples will be ready for in depth phenotypic analysis.

## Limitations

This protocol has been optimized for simultaneous detection and deep phenotyping of granulocyte populations (basophils, eosinophils, and neutrophils). Most antibodies used were conjugated to metal isotopes in house. Thus, this protocol requires a basic level of expertise in antibody panel design and validation of antibody conjugations.

The main limitation for this protocol is that samples must be stained fresh with surface antibodies. This prerequisite adds hours of labor, as the staining cannot be applied to a batch of samples that have been previously fixed. For optimal distinction between eosinophils and neutrophils, samples must first undergo RBC lysis, surface staining, and ultimately fixation with PFA (Figure 1A). We stress the importance of surface staining fresh samples because CD16 expression in eosinophils appears to be overrepresented when samples are fixed prior to staining with surface antibodies (Figures 1B and 1C). Therefore, this protocol would not be ideal for eosinophil and neutrophil detection if cells have already been fixed prior processing for mass cytometry analysis. For example, this protocol would not be optimal for detecting phospho-signaling in eosinophils or neutrophils, as this process would require fixation shortly after immune stimulation. However, basophils can still be readily detected even if fixation occurs prior to surface staining.

## Troubleshooting

### Problem 1

We cannot distinguish eosinophils from neutrophils in our gating scheme (steps 16 and 52).

### Potential solution

We rely on CD16 expression to distinguish eosinophils from neutrophils (Figure 1A), and when CD16 is applied to fixed cells, we lose the characteristically lower levels of CD16 in eosinophils compared to neutrophils (Figures 1B and 1C). Thus, we recommend that CD16, which is part of our surface antibody stain, be applied on live cells only. Unfortunately, this makes sample processing more laborious because cells must be collected and stained within a short time frame.

### Problem 2

Antibodies conjugated in house failed to detect desired target protein (before you begin, step 1).

### Potential solution

Choose a carrier free antibody when doing metal conjugations. Protein stabilizers such as BSA, and to a lesser extent gelatin, are rich in cysteine, which will compete with for free maleimide groups of the Maxpar polymer used for metal conjugation.

Furthermore, when purchasing primary antibodies for in house conjugation, choose an antibody clone that has been verified to work by flow cytometry. All antibody clones listed in our Resource Table were either confirmed to work by flow cytometry by the vendor or were rigorously tested in house against antibody markers with known specificity. If the desired antibody target does not have a flow cytometry specific clone, due diligence must be carried out to test its efficacy by CyTOF.

### Problem 3

Less than 50,000 events were captured by the CyTOF for a given sample (steps 5 and 29).

### Potential solution

If starting out with 1 mL of whole blood, or around 10 million cells post red blood cell lysis, the expected collection by CyTOF should be at least 500,000 events. While it is unnecessary to collect all events in the sample, enough events (around 100,000) are needed to conduct downstream analysis on rare populations like basophils and their subpopulations.

There are many steps in the protocol that are designed to retain the highest number of cells for analysis. Primarily, washing steps can greatly reduce cell counts. We suggest leaving some supernatant to avoid aspirations of cells in the pellet post centrifugation. Other steps where cell counts can be negatively impacted include the red blood cell (RBC) lysis and methanol permeabilization steps. These steps should be conducted at 4°C and carrying them out at 20°C–23°C with our suggested timing could lead to excessive cell death of white blood cells during RBC lysis, and loss of cell integrity during methanol permeabilization.

### Problem 4

The 24-h refrigeration period of whole blood does not work for our pipeline (step 1).

### Potential solution

Our blood samples were refrigerated for 24 h prior to processing mainly for convenience. Mukai et al. (2017) explore different blood storage conditions and their effect on basophil activation. The amplitude of the activation response as measured by CD203c and CD63 detection appear to decline with longer storage prior to processing. We recommend that blood storage conditions are kept consistent across samples. We did not evaluate the effect of blood storage on basophil subpopulations or other granulocyte phenotypes.

### Problem 5

We cannot detect granulocytes in our blood samples (step 5).

### Potential solution

Ficoll is commonly used to process peripheral blood samples and isolate peripheral blood mononuclear cells (PBMCs). Granulocytes have multi-lobed nuclei and Ficoll is typically used to eliminate them from the sample. Therefore, we recommend RBC lysis to process peripheral blood and retain granulocytes.

Additional steps must be taken to preserve the integrity of granulocytes in the samples. We use a low centrifugation speed (350 × *g* maximum) when spinning live cells, and we only switch to 500 × *g* in post fixation steps. Furthermore, when cell pellets must be resuspended, we gently tap the tube instead of vortexing or pipetting up and down. We suggest these steps to avoid disrupting granulocytic cells, which are particularly fragile.

## Resource availability

### Lead contact

Further information and requests for resources and reagents should be directed to and will be fulfilled by the lead contact, Dr. Sean Bendall (bendall@stanford.edu).

### Materials availability

This study did not generate new unique reagents.

## Data Availability

All relevant data and code are available from authors upon request.
